# Prevalence of erectile dysfunction and associated factors among diabetic men attending the diabetic clinic at Felege Hiwot Referral Hospital, Bahir Dar, North West Ethiopia, 2016

**DOI:** 10.1186/s13104-018-3211-2

**Published:** 2018-02-15

**Authors:** Bizuayehu Walle, Kidist Reba Lebeta, Yamrot Debela Fita, Hordofa Gutema Abdissa

**Affiliations:** 1 Department of Physiology, College of Medicine and Health Sciences, Bahir Dar University, Bahir Dar, Ethiopia; 2 School of Nursing, College of Medicine and Health Sciences, Bahir Dar University, Bahir Dar, Ethiopia; 3 Department of Health Education and Behavioural Sciences, School of Public Health, College of Medicine and Health Sciences, Bahir Dar University, Bahir Dar, Ethiopia

**Keywords:** Erectile dysfunction, Diabetes mellitus, Bahir Dar, Ethiopia

## Abstract

**Objective:**

Even though several scholars have conducted study in different part of the world on erectile dysfunction in patients diagnosed with diabetes mellitus, it’s magnitude vary among their finding with the range between 20 and 90%. This study aimed at assessing the prevalence of erectile dysfunction and associated factors among diabetic clients.

**Results:**

A cross sectional study was conducted from January 2016 to March, 2016. Systematic random sampling technique was used. Data were collected using a structured questionnaire and level of erectile dysfunction was measured using the international index of erectile function. A total of 422 diabetic patients were participated with 100% response rate. The proportion of erectile dysfunction was 85.5% and it was significantly associated with higher age (AOR: 6.46, 95% CI 2.55–16.44) and Diabetic complication (AOR: 3.97, 95% CI 1.06–17.36). Therefore, screening for ED in diabetic patients, particularly for those who are in advanced age and living with DM for more than 10 years is needed for it’s early detection, prevention and management.

## Introduction

The effective hydraulic action of the penis is a very important for practicing sexual intercourse and for the continuation of life. However, getting erection of the penis can be affected by various endogenous and exogenous factors. Inability of a person to get sufficient penile erection during sexual intercourse due to these factors may results a disease called erectile dysfunction (ED). Patients who are diagnosed with diabetes mellitus (DM) mostly experience such kind of abnormality [1].

In a scientific explanation, there are organic and a psychogenic type of causes of erectile dysfunction and the organic causes of ED has multiple categories such as: hormone-induced, drug-induced, vascular, traumatic/post-surgical, and neurological. Psychogenic includes depression, relationship problems, and performance anxiety [2]. Researches confirmed that inability to get maximum erection of the penis related with different types of illnesses. However, living with the DM has been reported as the main cause for ED [3, 4].

Erectile dysfunction is known to be one of the pressing problems faced by people with diabetes. A study result shows that 75% of men who live with diabetes are exposed to erectile dysfunction in their earlier ages as compared to non-diabetic men. Even if the risk of erectile dysfunction increases with age for all men, it appears to be higher among the diabetic men [5, 6].

Even though several scholars have conducted study in different part of the world on ED among DM patients, it’s magnitude vary among their finding with the range between 20 and 90%. Additionally, other researchers have presented individual engagement in unhealthy lifestyle and behaviour like smoking cigarette, alcohol and inactivity as determinant factor for ED [7–16]. Such discrepancy will make the understanding of the exact magnitude of ED among DM patients.

In Ethiopia, chronic medical illnesses are alarmingly raising with their complications. But, there is no any study, particularly on ED among DM patients although it has been reported as the common complication of DM. Thus, knowing it’s current magnitude is very important and can be used as evidence for early detection, management and improving DM patient’s quality of life through prevention of the psychologic impacts. Hence, this research undertook aiming at investigating the prevalence of erectile dysfunction and associated factors of diabetic men who are currently following up at Felege Hiwot Referral Hospital (FHRH).

## Main text

### Study design and participants

A cross sectional study design was employed in Felege Hiwot Referral Hospital from January to March, 2016. FHRH is found in Bahir Dar city, the capital of Amhara region in northwest of Ethiopia. Adult men diagnosed with diabetes and who are receiving pharmacological treatment for the disease were included in the study after excluding those who are seriously ill during data collection, individuals with ED before the diagnosis of diabetes and those not engaging in any form of sexual activity at the time of the interview.

### Sample size determination

The sample size was calculated using single population proportion formula. Assuming, proportion of erectile dysfunction as 50% since no previous study was conducted in the study area, confidence interval was 95% and marginal error was 5%. The final sample size was determined to be 422 by adding 10% non-response rate. The study participants were selected from using systematic sampling methods after developing the sampling frame from the FHRH’s follow up clinic register, the sampling interval was calculated by dividing the number of individuals in the frame by the sample size. Thus, since the calculated sampling interval was six, the data was collected from selected study participants with interval every five of patients diagnosed with diabetes visiting the follow up clinic each working days during the study period.

### Operational definitions

#### Erectile dysfunction

The presence of erectile dysfunction was established by using the international index of erectile function (IIEF-5) [11]. Individuals who scored 1–21 out of 25 points were reported as having ED. While those who scored 22–25 out of 25 points were reported as not having ED. Those who scored 1–7, 8–11 and 12–21 out of 25 points were classified as severe ED, Moderate ED and Mild ED respectively.

### Data collection procedure

The data was collected using an interview administered structured questionnaire and by reviewing the document. The questionnaire comprised of socio-demographic characteristics, DM related questions and international index of erectile function (IIEF-5) [11]. Three data collectors and one supervisor who are health professional were recruited. Two-day training was given for data collectors and supervisor on the procedure they have to follow during the data collection. Pretested was done on 5% of sample size at Debre Markos referral hospital on DM patient and corrections on some part of instrument was made based on the finding. On daily basis, during the data collection period, the collected data was checked for completeness and accuracy.

### Statistical analysis

The data was entered into Epi data 3.1 and analysed using SPSS version 20. Data cleaning and assumption checking were performed prior to proceeding for analysis. Descriptive statistical analysis like frequency and percentage for the categorical variables and mean, standard deviation and percentage for continues variables were done. The Chi square test used to check the association between dependent and independent variables. Multiple logistic regression analysis was employed to determine whether independent factors predict erectile dysfunction. The result of the OR was used for interpretation of strength of prediction of the independent variables to the outcome. For all statistical significance tests, the cut-off value set was p < 0.05 with confidence interval of 95%.

### Result

#### Demographic characteristics and medical condition

Making the response rate 100%, all of 422 selected individuals were participated in this study. Table 1 shows the socio demographic characteristic of the study participant. Accordingly, the mean age of the participants was 45.7 years old. Majority (73.7%) of the respondents were married. Out of the total respondents, 70.9% were orthodox Christian religion followers. Almost one-third (33.9%) of participated completed their college education. More than half (58.5%) of the participants were government employees.

**Table 1:** Socio-demographic characteristics of diabetic men attending the diabetic clinic at Felege Hiwot Referral Hospital Bahir Dar, Ethiopia (n=422)

Variable	Frequency (N)	Percent (%)
Age (years)		
18–30	106	25.1
31–44	93	22
45–59	118	28
≥ 60	105	24.9
Marital status		
Single	90	21.3
Married	311	73.7
Others^a^	21	5
Religion		
Orthodox	299	70.9
Muslim	104	24.6
Protestant	19	4.5
Education status		
No formal education	112	26.5
Primary school completed	130	30.8
High school completed	37	8.8
College and above	143	33.9
Ethnicity		
Amhara	385	91.2
Others^b^	37	8.8
Occupation		
Unemployed	175	41.5
Employed	247	58.5
Monthly income (ETB)		
< 1000	80	19
> 1000	342	81

*ETB* Ethiopian Birr

^a^Others (widowed and divorce), ^b^ others (Oromo, Gurage and Tigray)

#### Erectile dysfunction and medical condition

The prevalence of erectile dysfunction and medical conditions is presented on Table 2. More than four out of five (85.5%) study participants have experienced erectile dysfunction. Of those who experience erectile dysfunction, majority (64.8%) of them have moderate of dysfunction. Majority (65.4%) of the respondents live with diabetes for < 5 years with mean duration of 5.2 years. Majority (59%) of respondents used intravenous insulin injection medication only. Less than one-third (28.4%) of the study participant had developed diabetic complication. Of those individual who developed diabetic complication, 72 (60%) of them had developed cardiovascular disorder while only 7 (6%) of them had foot ulcer.

**Table 2:** Erectile dysfunction and medical conditions of diabetic men attending the diabetic clinic at Felege Hiwot Referral Hospital Bahir Dar, Ethiopia (n=422)

Variable	Frequency	Percent (%)
Duration of diabetes (years)		
≤ 5	274	64.9
6–10	94	22.3
> 10	54	12.8
Treatment for diabetes		
Oral medications only	123	29.2
Insulin only	249	59.0
Insulin and oral medication	50	11.8
Diabetic complication		
No	302	71.6
Yes	120	28.4
Type of complication (n = 120)		
Renal disease	16	13
Cardiovascular disease	72	60
Eye complication	25	21
Foot ulcer	7	6
Erectile dysfunction		
Yes	361	85.5
No	61	14.5
Category of ED (n = 361)		
Mild	66	18.3
Moderate	234	64.8
Severe	61	16.9

#### Factors associated with erectile dysfunction

Table 3 shows the factors associated with erectile dysfunction. Age and duration of diabetes were the only factors associated with erectile dysfunction. Men who were in age group of 45–59 years and those who aged 60 and above years were 6.5 and 7 times more likely experienced erectile dysfunction as compared to those in age group of 18–30 years old (AOR: 6.46, 95% CI 2.55–16.44) and (AOR: 7.14, 95% CI 2.61–19.45) respectively. Men who were living with DM for more than 10 years were four times more likely experienced erectile dysfunction as compared with those who are living with it for 5 years and less with (AOR: 3.97, 95% CI 1.06–17.36).

**Table 3:** Factors associated with erectile dysfunction among male diabetic clients attending diabetic clinic at Felege Hiwot Referral Hospital Bahir Dar, Ethiopia (n = 422)

Variable	Erectile dysfunction		Odd ratio (95% CI)	
	Yes	No	Crude	Adjusted
Age (years)				
18–30	76	30	1	1
31–44	73	20	1.44 (0.75–2.76)	1.49 (0.77–2.88)
45–59	112	6	7.37 (2.93–18.56)*	6.46 (2.55–16.44)*
≥ 60	100	5	7.89 (2.93–21.30)*	7.14 (2.61–19.45)*
Marital status				
Single	65	25	1	1
Married	278	33	3.24 (1.80–5.82)*	2.36 (0.91–4.66)
Others	16	5	2.31 (0.48–11.05)	1.67 (0.31–8.95)
Duration of diabetes (years)				
≤ 5	222	52	1	1
6–10	87	7	2.91 (1.27–6.66)*	2.61 (1.11–6.12)*
> 10	46	8	6.09 (1.44–25.81)*	3.97 (1.06–17.36)*
Diabetic complication				
No	246	56	1	1
Yes	115	5	5.24 (2.04–13.42)*	2.14 (0.76–6.03)

italic and *Significant at *p* value < 0.05

### Discussion

In this study, the magnitude of erectile dysfunction was 85.5% which is higher than previous studies in Egypt (63.6%), Nigeria (57.4%), Jordanian (62%), and Jamaica (64%) [14, 17, 18]. However, the proportion is lower than a study conducted in Pakistan 88% [14]. This difference might be attributable to differences in among the study population, the methodology used, time of study and different population culture.

The finding of the current study showed that age was significantly associated with ED. Diabetic patients who were in age group of 45–59 years and those who aged 60 and above years were 6.5 and 7 times more likely experienced erectile dysfunction as compared to those in age group of 18–30 years old (AOR: 6.46, 95% CI 2.55–16.44), (AOR: 7.14, 95% CI 2.61–19.45) respectively. Similar study conducted in Tanzania, ED was significantly predicted by old age (odds ratio (OR) = 7.1, 95% CI 1.2–40.7) [19]. Another similar study conducted in Jamaica, disclosed that the prevalence of ED increased from 36% in 50–59 years age group to 90% in 70–75 years age group [18]. In other studies, age was perhaps predicted ED in diabetic men as well as in the general population [14, 20].

In this study, duration of DM is significantly associated with ED. Men who were living with DM for more than 10 years were four times more likely experienced erectile dysfunction as compared with those who are living with it for 5 years and less (AOR: 3.97, 95% CI 1.06–17.36). In previous studies done in Saudi, it was also reported that men who lived with DM for more than 10 years had experienced ED than those who had history of < 5 years of living with DM [20].

### Conclusion

The finding of current study revealed high prevalence of erectile dysfunction on male DM patients. Majority of DM patients experienced moderate erectile dysfunction. Old age and living with DM for more than 10 years were significant predictor of ED. Therefore, screening for ED for diabetic patients particularly, for those who aged above 45 years and living with DM for more than 10 years is needed for early detection, treatment and possibly prevention.

### Limitations

Since this study have used cross-sectional design, we cannot report cause and effect. Future study should examine the causal relationship of the variables using analytical study design. The issue of social desirability bias should also be considered in current study.

## Abbreviations

AOR: adjusted odd ratio
DM: diabetes mellitus
ED: erectile dysfunction
FHRH: Felege Hiwot Referral Hospital
IIEF: international index of erectile function
OR: odd ratio
SPSS: statistical package for social sciences
